# What can the neurological manifestations of COVID-19 tell us: a meta-analysis

**DOI:** 10.1186/s12967-021-03039-2

**Published:** 2021-08-23

**Authors:** Yuanyuan He, Xiaojie Bai, Tiantian Zhu, Jialin Huang, Hong Zhang

**Affiliations:** Department of Emergency Medicine, The First Affiliated Hospital of Anhui Medical University, 218 jixi road, shushan district, Hefei, Anhui China

**Keywords:** COVID-19, SARS-CoV-2, Neurologic manifestations, The prevalence rate

## Abstract

**Background:**

Covid-19 became a global pandemic in 2019. Studies have shown that coronavirus can cause neurological symptoms, but clinical studies on its neurological symptoms are limited. In this meta-analysis, we aimed to summarize the various neurological manifestations that occurred in COVID-19 patients and calculate the incidence of various neurological manifestations. At the same time, we further explored the mechanism of nervous system injury and prognosis in COVID-19 patients in combination with their nervous system manifestations. This study provides a reference for early clinical identification of COVID-19 nervous system injury in the future, so as to achieve early treatment and reduce neurological sequelae.

**Methods:**

We systematically searched all published English literature related to the neurological manifestations of COVID-19 from January 1, 2020, to April 30, 2021, in Pubmed, Embase, and Cochrane Library. The keywords used were COVID-19 and terminology related to the nervous system performance. All included studies were selected by two independent reviewers using EndNote and NoteExpress software, any disagreement was resolved by consensus or by a third reviewer, and the selected data were then collected for meta-analysis using a random-effects model.

**Results:**

A total of 168 articles (n = 292,693) were included in the study, and the meta-analysis showed that the most common neurological manifestations of COVID-19 were myalgia(33%; 95%CI 0.30–0.37; I^2^ = 99.17%), smell impairment(33%; 95%CI 0.28–0.38; I^2^ = 99.40%), taste dysfunction(33%; 95%CI 0.27–0.39; I^2^ = 99.09%), altered mental status(32%; 95%CI 0.22–0.43; I^2^ = 99.06%), headache(29%; 95%CI 0.25–0.33; I^2^ = 99.42%), encephalopathy(26%; 95%CI 0.16–0.38; I^2^ = 99.31%), alteration of consciousness(13%; 95%CI 0.08–0.19; I^2^ = 98.10%), stroke(12%; 95%CI 0.08–0.16; I^2^ = 98.95%), dizziness(10%; 95%CI 0.08–0.13; I^2^ = 96.45%), vision impairment(6%; 95%CI 0.03–0.09; I^2^ = 86.82%), intracerebral haemorrhage(5%; 95%CI 0.03–0.09; I^2^ = 95.60%), seizure(4%; 95%CI 0.02 -0.05; I^2^ = 98.15%), encephalitis(2%; 95%CI 0.01–0.03; I^2^ = 90.36%), Guillan-Barré Syndrome (GBS) (1%; 95%CI 0.00–0.03; I^2^ = 89.48%).

**Conclusions:**

Neurological symptoms are common and varied in Covid-19 infections, and a growing number of reports suggest that the prevalence of neurological symptoms may be increasing. In the future, the role of COVID-19 neurological symptoms in the progression of COVID-19 should be further studied, and its pathogenesis and assessment methods should be explored, to detect and treat early neurological complications of COVID-19 and reduce mortality.

## Introduction

At the end of December 2019, an epidemic of COVID-19 occurred in Wuhan, Hubei Province, China. As the epidemic has spread, cases have been found in many countries. At the time of writing, it has spread to more than 200 countries and regions, with a total of 187,637,579 confirmed cases and 4,066,292 death. Coronavirus has caused two fatal outbreaks in the past, the first in China in 2003, with a 10 percent mortality rate; The second outbreak was Middle East Respiratory Syndrome (MERS) in Saudi Arabia in 2012, with a mortality rate of 35% [1, 2]. Novel Coronavirus was named severe acute respiratory syndrome coronavirus Type 2 (SARS-COV-2). Sars-Cov-2 is a single-stranded RNA coronavirus, belonging to the same -coronavirus branch as SARS-COV and MERS-CoV. Its genome sequence is 89.1% similar to the nucleotide sequence of a group of SARS-like coronaviruses. Studies have found that the viral structure and receptor binding domain of SARS-COV-2 is similar to that of SARS-COV, and both of them enter human cells by binding to ACE2 receptors [3, 4]. To evaluate the genetic variation of SARS-COV-2, 86 complete or nearly complete genomes were genetically analyzed, and many mutations and deletions were found in both coding and non-coding regions [5]. At present, the biological characteristics and genetic variation of SARS-COV-2 are still not very clear and deserve further study.

Although COVID-19 is typically characterized by respiratory symptoms, numerous clinical studies have shown that patients infected with SARS-COV-2 are associated with acute injuries to external pulmonary organs, including the heart, digestive tract, liver, kidney, and nervous system [6, 7]. More and more clinical evidence indicates that SARS-COV-2 may invade the central nervous system. A retrospective analysis of 214 patients with COVID-19 found that 78 (36.4%) had nervous system involvement, and 28.2% of them had severe central nervous system injury [8]. Studart-Neto A et al. retrospectively analyzed 1208 patients with COVID-19 and found that 89 (7.4%) presented neurological manifestations, including encephalopathy (44.4%), stroke (16.7%), epilepsy (9.0%), neuromuscular disease (5.6%), another acute brain injury (3.4%), and other mild non-specific diseases (11.2%) [9]. Takeshi et al. detected SARS-COV-2 RNA in cerebrospinal fluid specimens, providing direct evidence for the nervous invasiveness of SARS-COV-2 [10]. There are three main types of COVID-19 nervous system involvement: (1) central nervous system involvement, such as dizziness, headache, disturbance of consciousness, acute cerebrovascular disease, and epilepsy; (2) peripheral nervous system involvement, including anosmia, decreased taste, decreased vision, and neuralgia; (3) skeletal muscle injury [11, 12].COVID—19 possible mechanisms include: nerve injury by angiotensin-converting enzyme (ACE2) receptor function, blood and break through the blood–brain barrier, through the way such as the olfactory nerve attack the nervous system [13, 14].

Since nervous system manifestations are an important part of COVID-19 patients' clinical manifestations, early detection and treatment of nerve injury in COVID-19 patients is of great significance for their prognosis and reduction of neurological sequelae. This study aims to further summarize the incidence and characteristics of various nervous system manifestations in COVID-19 patients, and explore the mechanism and prognosis of nervous system injury in combination with nervous system manifestations, so as to provide positive clinical significance for better epidemic prevention and control and clinical diagnosis and treatment in the future.

## Methods

### Search strategy and research selection

We searched the following electronic databases for literature published between 1 January 2020 and April 30, 2021: Pubmed, Embase and the Cochrane Library. The search terms used include: ((severe acute respiratory syndrome coronavirus 2) OR (SARS-CoV-2) OR (2019-nCoV) OR (2019 novel coronavirus) OR (COVID-19) OR (coronavirus) OR (novel coronavirus pneumonia) OR (NCP)) AND ((neurologic manifestations) OR (neurological manifestations) OR (neurologic manifestation) OR (neurologic signs and symptoms) OR (neurological manifestation) OR (neurologic deficits) OR (neurologic deficit) OR (neurologic symptoms) OR (neurologic symptom) OR (neurologic findings) OR (neurologic finding) OR (neurologic signs) OR (neurologic sign) OR (neurologic dysfunction) OR (neurologic dysfunctions) OR (nervous system diseases) OR (nervous system disease) OR (neurologic disorders) OR (neurologic disorder) OR (neurological disorders) OR (neurological disorder) OR (nervous system disorders) OR (nervous system disorder) OR (neurological complication) OR (neurological complications) OR (nerve function injury)).

### Inclusion and exclusion criteria

The inclusion criteria for this study are as follows: all English literature from 1 January 2020 solstices 30 April 2021 that has been published reporting neurological manifestations of COVID-19 patients. Studying types include case–control studies, cohort studies, cross-sectional studies, and case series. Only those subjects who were diagnosed with SARS-COV-2 infection by real-time reverse transcription-polymerase chain reaction or by high-throughput sequencing of swab samples were included in the study. Only those studies whose results included specific neurological manifestations were included.

Exclusion criteria included: (1) no clinical manifestations of related neurological symptoms and lack relevant data; (2) Age < 18 years old; (3) Repeated studies; (4) Editorials, reviews, systematic reviews, meta-analyses, comment, animal studies, postmortem studies, etc. (5) Documents not written in English.

### Data extraction and quality assessment

A comprehensive and rigorous review of all literature retrieved was conducted by two independent study members according to inclusion and exclusion criteria, and any disagreement between reviewers was determined by consultation between the two or by a third investigator. Extracting data from each study include the first author's name, year, country, research design, time and duration, participants details (gender, age, the overall sample size and specific symptoms of patients number) and neurologic manifestations, such as headache, dizziness, altered mental status, stroke, intracerebral hemorrhage, seizure, myalgia, smell impairment, taste dysfunction, vision impairment, alteration of consciousness, encephalitis, encephalopathy and Guillan-Barré Syndrome (GBS).

The STROBE Statement was used to evaluate the quality of the included studies. It consists of 22 projects and is used to evaluate six main components, including title and abstract, Introduction, Methods, Results, Discussion, and other information. Quality evaluations were conducted by two independent researchers, and any differences between them were resolved by discussion or by the intervention of a third researcher.

### Statistical analysis

Our study synthesizes the results of several similar studies to provide a quantitative summary. The extracted data details and instructions are presented in tables and figures. We used Stata (version 15.1) for the meta-analysis. I^2^ was calculated to assess the level of heterogeneity and can be divided into four categories: maybe unimportant (0–40%), may represent moderate heterogeneity (30–60%), may represent significant heterogeneity (50–90%) and significant heterogeneity (75–100%). In this study, a fixed-effect model or random-effect model was used to calculate the comprehensive prevalence rate and 95% confidence interval. When there was no heterogeneity, the fixed effect model was selected; when there was heterogeneity, a random effect model was selected. Forest maps are used to visually indicate the magnitude of the effects that the study summarizes and their 95% confidence intervals. Funnel plot and Egger's test were used to assess the publication bias of all literature, and P < 0.05 was considered statistically significant.

## Results

**Fig. 1:**
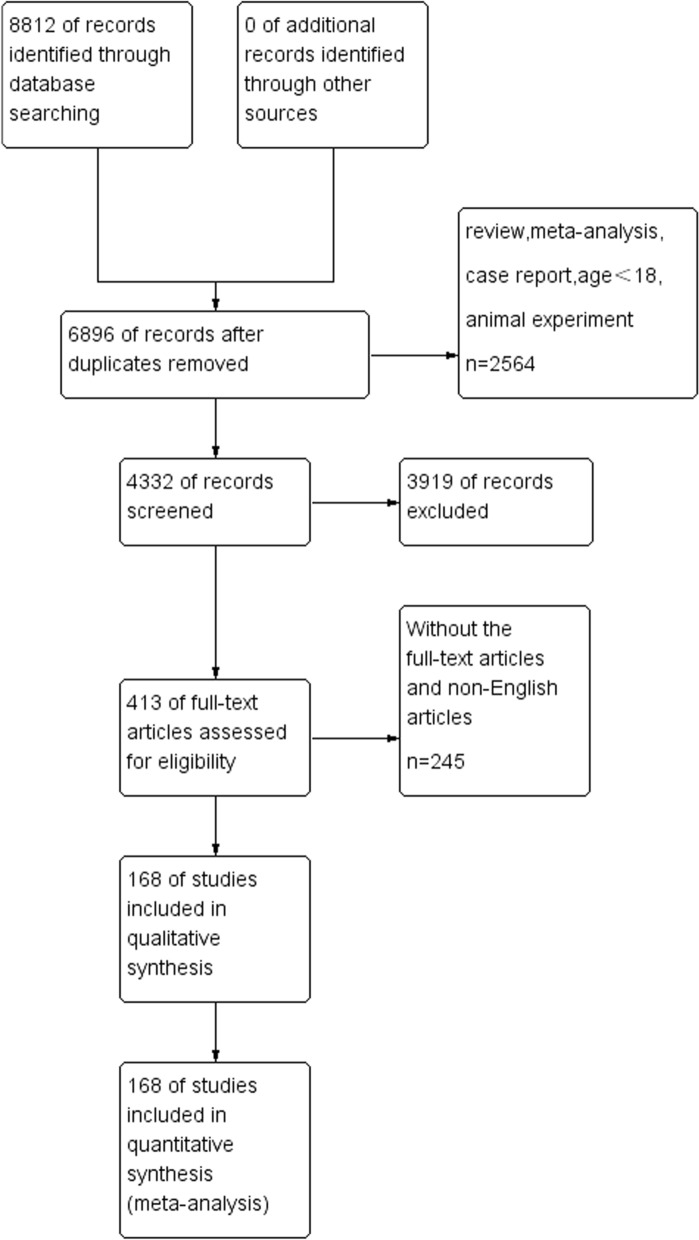
The Study flow Diagram

A total of 8812 studies were selected from the database, including 5789 in Pubmed, 2971 in Embase, and 52 in the Cochrane Library. After eliminating duplicate studies (n = 1916), 6896 studies met the preliminary screening criteria based on titles and abstracts. After excluding review, mata analysis, case report, age < 18, animal experiment and literature with inconsistent research contents (n = 6483), the remaining 413 articles were screened according to the inclusion and exclusion criteria. After the full-text screening, 168 articles (N = 292,693) were included in the meta-analysis. Figure 1 showed the Study Flow Diagram. All of the studies were published in 2020 and 2021. Among the included studies, 6 studies were case–control studies,10 studies were case series, 13 were cohort studies, and 25 were cross-sectional studies.104 retrospective studies, 36 prospective studies and 29 multicenter studies were included. Major countries included were 29 studies in the USA, 20 in Italy, 17 in Spain, 15 in France, 15 in China and 12 in Turkey. Table 1 showed the details and characteristics of the included studies.

**Table 1:** The details and characteristics of the included studies

Study	Design	Country	Case	Male(%)	Age (mean)	Neurologic manifestations													
						Headache	Dizziness	Altered mental status	Intracerebral haemorrhage	Seizure	Stroke	Myalgia	Smell impairment	Taste dysfunction	Vision impairment	Alteration of consciousness	Encephalopathy	Encephalitis	Guillan-Barré Syndrome (GBS)
José Manuel Abalo-Lojo et al. [15]	RS	Spain	131	42.6	50.4	51	8	/	/	/	/	61	77	74	/	/	/	/	/
Türkan Acar et al. [16]	RS	Turkey	30	43.3	51.6	10	3	/	/	/	/	15	10	/	/	/	/	/	/
Pinky Agarwa et al. [17]	RS	USA	404	48.8	61	82	31	86	/	2	3	131	18	27	5	/	/	/	/
H Avc et al. [18]	RS	Turkey	1197	58.5	49.07	173	/	/	/	/	/	257	529	526	/	/	/	/	/
Ameen Biadsee et al. [19]	CS	Israel	128	45.3	36.25	52	/	/	/	/	/	60	49	42	/	/	/	/	/
Aylin C¸alıca Utku et al. [20]	RS	Turkey	143	53.9	55.63	43	/	/	/	/	/	49	33	51	/	/	/	/	/
Edoardo Caronna et al. [21]	PS	Spain	130	49.2	53.9	97	19	/	/	/	/	39	/	/	/	/	/	/	/
Eleonore Chary et al. [22]	PS,CS *	France	115	30	47	62	/	/	/	/	/	57	/	/	/	/	/	/	/
Yanan Li et al. [23]	RS	China	219	40.6	53.3	/	/	/	1	/	10	/	/	/	/	/	/	/	/
Aaron Rothstein et al. [24]	RS	USA	844	48	59	/	/	/	8	/	20	/	/	/	/	/	/	/	/
James E Siegler et al. [25]	RS,cohort study*	USA	172	59.5	69.5	/	/	/	28	/	156	/	/	/	/	/	/	/	/
Prof Stéphane Kremer et al. [26]	RS *	France	37	81	61	4	/	/	/	5	/	/	/	/	/	27	/	/	/
Veria Vacchiano et al. [27]	Questionnaire	Italy	108	57	59	46	11	/	/	/	/	37	40	66	/	/	/	/	/
Ot´avio Melo Esp´ındola et al. [28]	CS	Brazil	58	43.5	51.6	14	/	/	6	/	/	/	/	/	/	/	24	/	6
Jerome R. Lechien et al. [29]	Questionnaire	Europe	1420	32.3	39.17	998	/	/	/	/	/	887	997	770	/	/	/	/	/
Imen Kacem et al. [30]	RS	Tunisia	646	53.9	42.17	279	/	/	/	/	/	241	245	238	48	/	/	/	/
B.C. Yoon et al. [31]	RS	USA	26	61.5	63.6	2	/	14	11	1	2	/	/	/	/	/	/	/	/
Chaolin Huang et al. [32]	PS	China	41	73	49	3	/	/	/	/	/	18	/	/	/	/	/	/	/
Manuel Requena et al. [33]	PS	Spain	2050	56	66.5	/	/	/	4	/	21	/	/	/	/	/	/	/	/
Jennifer A. Frontera et al. [34]	PS *	USA	4491	66	71	/	/	/	/	74	84	/	/	/	/	/	309	/	3
Timothée Klopfenstein et al. [35]	RS	France	54	33	47	44	/	/	/	/	/	40	54	/	4	/	/	/	/
Maryam Jalessi et al. [36]	PS	Iran	92	67.4	52.94	20	/	/	/	/	/	57	22	15	/	/	/	/	/
David García-Azorín et al. [37]	CSS	Spain	104	36.5	56.7	104	3	10	/	/	/	44	67	/	/	6	/	/	/
Samar Iltaf Sr et al. [38]	CSS	PAK	350	70	49.5	21	12	/	/	1	/	/	5	/	/	7	/	3	1
Aamir Makda et al. [39]	CSS	PAK	114	54.4	51	18	20	/	/	1	/	/	9	9	2	10	/	/	/
Pedro Augusto Sampaio Rocha-Filho et al. [40]	CSS	Brazil	73	63	58	47	/	/	/	/	/	/	28	29	/	/	/	/	/
Abdelkader Mahammedi et al. [41]	RS *	Italy	108	64	69	13	4	64	6	10	34	13	2	/	/	/	/	/	/
Antoine Guilmot et al. [42]	PS	Belgium	15	80	62	/	/	/	/	2	/	/	2	/	/	/	/	/	/
Jerome R. Lechien et al. [43]	RS *	Belgium	2013	34	39.5	1411	/	/	/	/	/	1244	1754	1136	/	/	/	/	/
Sara Mariotto et al. [44]	RS	Italy	107	76.6	65.8	12	6	/	/	/	/	16	15	22	/	13	/	/	/
Claudio Liguori et al. [45]	PS	Italy	103	57.3	55	40	27	/	/	/	/	25	40	48	/	/	/	/	/
Antoniangela Cocco et al. [46]	Questionnaire	Italy	78	52.6	53.7	52	34	/	/	/	/	/	/	/	25	/	/	/	/
Nao Yan et al. [47]	RS, cohort study	China	1682	48.9	50	216	15	/	/	/	/	311	/	/	/	/	/	/	/
Elodie Meppiel et al. [48]	RS *	France	222	61.3	65	24	5	117	5	21	52	/	7	4	/	/	67	21	15
Hisham Salahuddin et al. [49]	RS *	USA	574	48.1	62.83	82	40	/	/	5	7	80	37	43	/	/	143	/	/
Rishu Garg et al. [50]	CSS	India	106	61	49.07	24	20	/	/	/	/	20	26	35	/	/	/	/	/
Adalberto STUDART-NETO et al. [9]	RS	Brazil	89	61.8	57.4	3	2	/	2	4	11	18	8	3	/	35	40	/	/
M Petrocelli et al. [51]	Cohort study	Italy	301	25	43.6	133	/	/	/	/	/	128	141	114	/	/	/	/	/
Daniel J. Lee et al. [52]	CSS	Toronto	56	41.1	38	10	/	/	/	/	/	/	31	32	/	/	/	/	/
Yujie Liang et al. [53]	RS	China	86	51.2	25.5	12	/	/	/	/	/	8	34	33	/	/	/	/	/
Timothée Klopfenstein et al. [54]	RS	France	70	41	57	51	/	/	/	/	/	41	37	34	3	/	/	/	/
Weixi Xiong et al. [55]	RS,cohort study*	China	917	55	48.7	2	/	/	/	/	10	/	/	/	/	25	/	/	/
Jerome R. Lechien et al. [56]	PS *	Europe	417	36.9	36.9	/	/	/	/	/	/	/	357	342	/	/	/	/	/
Yonghyun Lee et al. [57]	RS	Korea	3191	36.4	44	/	/	/	/	/	/	/	389	353	/	/	/	/	/
Ömer Karadaş et al. [58]	PS	Turkey	239	55.6	46.46	64	16	/	/	/	/	36	18	16	8	23	/	/	1
Benoit Tudrej et al. [59]	CSS	France	816	35	45	359	/	/	/	/	/	166	156	188	/	/	/	/	/
Luigi Angelo Vaira et al. [60]	RS	Italy	72	37.5	49.2	30	/	/	/	/	/	/	14	9	/	/	/	/	/
Valeria Dell’Era et al. [61]	CSS	Italy	355	54.1	50	/	/	/	/	/	/	/	237	232	/	/	/	/	/
Luis Antonio Díaz et al. [62]	RS	Chile	7016	50	40	3040	/	/	/	/	/	3082	/	/	/	/	/	/	/
Ruth Levinson et al. [63]	RS	Israel	42	54.8	34	20	9	/	/	/	/	24	14	15	/	/	/	/	/
Paolo J. Fantozzi et al. [64]	RS,cohort study	Italy	111	52.3	57	/	/	/	/	/	/	3	46	66	/	/	/	/	/
Andy Jian Kai Chua et al. [65]	Cohort study	Singapore	31	/	/	/	/	/	/	/	/	/	14	/	/	/	/	/	/
Alvaro Beltrán-Corbellini et al. [66]	Case–control study *	Spain	79	60.8	61.6	/	/	/	/	/	/	/	25	28	/	/	/	/	/
Giacomo Spinato et al. [67]	RS	UK	202	48	56	86	28	/	/	/	/	/	/	/	/	/	/	/	/
Nitesh Gupta et al. [68]	PS	India	200	58	40.03	22	/	/	/	/	/	54	1	/	/	/	/	/	/
Marco Luigetti et al. [69]	RS	Italy	213	/	70.2	10	3	/	/	6	2	20	13	6	/	/	86	1	/
Erdal Sakalli et al. [70]	Questionnaire	Turkey	172	48.8	37.8	17	/	/	/	/	/	16	18	11	/	/	/	/	/
Eva Jiménez et al. [71]	RS,CS	Spain	1549	57.5	69	133	/	/	/	/	11	291	41	/	/	/	/	/	/
Yihui Huang et al. [72]	RS	China	34	41.2	56.24	2	/	/	/	/	/	/	/	/	/	/	/	/	/
Souheil Zayet et al. [73]	RS	France	70	41	57	51	/	/	/	/	/	41	37	34	3	/	/	/	/
Tyler Scullen et al. [74]	RS,CSS	USA	27	52	59.8	2	/	26	/	/	/	/	/	1	/	/	20	/	/
Ling Mao et al. [8]	RS,CS	China	214	40.7	52.7	28	36	/	/	1	/	/	11	12	3	16	/	/	/
Brad Tyson et al. [75]	RS	USA	71	47	79	5	9	47	/	3	2	11	/	/	/	/	/	/	/
Alex Carignan MD MSc et al. [76]	Case − control study	Canada	134	47.8	57.1	87	27	/	/	/	/	76	69	85	6	/	/	/	/
D. Hornuss et al. [77]	Case − control study	Germany	45	55.6	56	10	/	/	/	/	/	/	18	/	/	/	/	/	/
Firouzeh Heidari et al. [78]	CS	Iran	23	34.8	37.4	/	/	/	/	/	/	4	16	/	/	/	/	/	/
Kevin N. Sheth et al. [79]	PS,cohort study	USA	20	85	80	/	/	/	1	/	/	/	/	/	/	/	/	/	/
Carol H. Yan MD et al. [80]	CSS	USA	59	49.2	/	39	/	/	/	/	/	37	40	42	/	/	/	/	/
A. Mahammedi et al. [81]	RS *	USA	135	64	68.2	/	/	/	14	/	/	/	/	/	/	/	/	/	/
A. Radmanesh et al. [82]	RS, CS	USA	242	62	68.7	/	/	102	11	/	/	/	/	/	/	/	/	/	/
E. Lin et al. [83]	RS	USA	278	59	71.8	/	/	/	/	/	/	/	/	/	/	/	/	/	/
François Lersy et al. [84]	RS	France	58	66	62	3	/	/	/	6	/	/	/	/	/	/	47	/	/
Rajesh Benny et al. [85]	RS *	India	100	63	57	21	/	/	9	18	78	/	/	/	/	/	/	/	/
Deusdedit Branda ˜o Neto et al. [86]	PS	Brazil	655	35.3	37.7	143	/	/	/	/	/	/	539	502	/	/	/	/	/
Eman M. Khedr et al. [87]	RS, case–control study*	Egypt	439	54.5	62.8	/	/	/	/	/	53	/	/	/	/	/	/	/	/
Shin-Woo Kim et al. [88]	RS *	Korea	2254	35.8	58	378	/	/	/	/	/	385	27	/	/	24	/	/	/
Xiaolong Yao et al. [89]	RS	China	2474	49.9	61	/	/	/	/	/	25	511	/	/	/	66	/	/	/
G.-u. Kim et al. [90]	RS	Korea	172	66	26	54	32	/	/	/	/	54	68	58	/	/	/	/	/
Nathan J Brendish et al. [91]	PS,cohort study	UK	197	57.4	68	73	/	/	/	/	/	62	47	/	/	/	/	/	/
L. Cleret de Langavant et al. [92]	RS	France	26	73.1	58.3	11	/	/	/	/	4	/	/	/	/	/	6	8	2
Carlos DF de Souza et al. [93]	RS,CSS	Brazil	9807	47.5	70.21	1980	/	/	/	/	/	868	/	/	/	/	/	/	/
Daniel Schonfeld et al. [94]	RS	Argentina	207,079	50	42.9	93,939	/	/	/	435	/	55,812	53,273	/	/	/	/	/	/
Raphael L. Tuma et al. [95]	RS,CS	Brazil	55	68	61.2	/	/	/	/	/	/	/	/	/	/	/	43	/	/
Souheil Zayet et al. [96]	RS	France	95	16.8	39.8	74	/	/	/	/	/	71	60	62	/	/	/	/	/
Souheil Zayet et al. [97]	RS	France	62	39	56	48	/	/	/	/	/	38	32	/	/	/	/	1	/
Seyed Hadi Samimi Ardestani et al. [98]	CSS *	Iran	311	71.7	47	45	/	/	/	/	/	104	207	129	/	/	/	/	/
Andrea Pilotto et al. [99]	RS	Italy	147	49	73.1	2	/	38	/	10	/	/	/	/	/	/	/	/	/
Anne Schneider et al. [100]	RS	Germany	87	44.8	37	26	/	/	/	/	/	/	44	/	/	/	/	/	/
Shaista Alam et al. [101]	RS	USA	17	58.8	61	/	/	/	2	3	11	/	/	/	/	/	/	/	/
Behnam Sabayan et al. [102]	CS	Iran	15	80	65	/	/	/	1	/	14	/	/	/	/	/	/	/	/
Ryan HW Cho et al. [103]	PS,CSS	China	83	57.8	36.4	/	/	/	/	/	/	/	39	36	/	/	/	/	/
Fan-Yun Lan et al. [104]	RS	USA	83	27.7	43.9	34	/	/	/	/	/	47	/	/	/	/	/	/	/
Nishanth Dev et al. [105]	RS, case–control study	India	55	58	36	/	/	/	/	/	/	/	53	41	/	/	/	/	/
N’dri Juliette Kadiane-Oussou et al. [106]	RS	France	55	58.2	68.5	25	/	/	/	/	/	39	17	18	2	/	/	/	/
Udo Zifko et al. [107]	RS	Austria	82	53.7	56	25	13	/	/	1	4	5	22	25	/	/	/	/	/
Hiroki Nakanishi et al. [108]	RS	Japan	60	55	51.4	13	3	/	/	/	/	/	19	18	/	/	/	/	/
Wang-Huei Sheng et al. [109]	PS	China	217	46.5	33	/	/	/	/	/	/	/	73	62	/	/	/	/	/
Caizheng Yu et al. [110]	RS	China	1663	50.4	64	/	/	/	/	/	/	57	/	/	/	/	/	/	/
Shenae Samuels et al. [111]	RS	USA	1537	47.4	51	219	/	/	/	6	/	439	/	/	/	60	/	/	/
Javier Trigo et al. [112]	RS	Spain	576	56.7	67.2	137	/	/	/	/	/	139	146	/	/	/	/	/	/
Eric M. Liotta et al. [113]	RS	USA	509	55.2	58.5	192	151	/	/	4	7	228	58	81	/	/	162	1	/
Javier A. Membrilla et al. [114]	CSS	Spain	45	37.8	40.4	39	/	/	/	/	/	23	28	/	/	/	/	/	/
Alfonso Coppola et al. [115]	PS	Italy	73	71.2	69.75	29	/	/	/	/	/	/	/	/	/	/	/	/	/
Sarah I.M. Janus et al. [116]	RS	Netherlands	88	27.3	83.5	17	/	/	/	1	/	/	/	/	/	/	/	/	/
Piotr Tekiela et al. [117]	RS *	USA	4491	/	/	/	/	/	/	539	629	/	/	/	/	/	2290	/	/
Alberto J. Guillén Martínez et al. [118]	RS	Spain	126	50	51.46	63	/	/	/	/	/	/	79	75	/	/	/	/	/
Aline Mendes et al. [119]	RS,CSS	Switzerland	265	43	85.9	/	/	/	/	/	11	/	/	/	/	/	/	/	/
Sara Radmard et al. [120]	RS,CS	USA	33	60.6	56.1	/	/	/	/	9	5	/	/	/	/	/	12	/	/
Russell R. Kempker et al. [121]	RS	USA	51	/	/	/	/	/	/	/	/	28	26	27	/	/	/	/	/
Patrick Dawson et al. [122]	RS	USA	42	53	21	32	/	/	/	/	/	24	18	24	/	/	/	/	/
Vanessa Oliveira et al. [123]	RS *	Portugal	1261	51.3	70	169	8	/	/	19	/	114	52	47	/	122	/	/	/
Stéphane Kremer et al. [124]	RS *	France	64	67	65	10	/	/	/	1	17	/	2	4	/	25	/	8	/
Nicola Rifino et al. [125]	RS	Italy	1760	66	64.9	3	/	49	/	10	37	/	/	/	/	/	/	5	17
Pria Anand et al. [126]	PS	USA	74	57	64	13	5	39	/	15	14	18	2	/	/	3	26	/	/
Fernando Daniel Flores-Silva et al. [127]	PS,CSS	Mexico	1072	65	53.2	76	4	11	/	9	9	75	11	14	/	/	/	2	/
Juan Carlos Garcia-Monco et al. [128]	PS	Spain	100	62	63.5	44	36	/	/	2	/	43	/	/	/	/	9	/	/
Carlos Manuel Romero-Sánchez et al. [129]	RS *	Spain	841	56.2	66.42	119	51	/	3	6	11	145	41	52	/	/	/	1	/
Emad Nader Eskandar et al. [130]	RS *	USA	4711	53.3	66.3	/	/	258	/	26	55	/	/	/	/	/	/	/	/
Aravinthan Varatharaj et al. [131]	PS	UK	153	48	71	/	/	39	9	/	57	/	/	/	/	/	9	7	/
Xudong He et al. [132]	RS	China	77	49.4	56	4	9	/	/	/	/	18	1	/	/	/	/	/	/
Suman Kushwaha et al. [133]	CSS	India	14	50	41.5	4	/	/	/	5	2	/	2	/	/	/	/	/	1
Alberto Romagnolo et al. [134]	RS	Italy	344	59.3	61.5	/	/	/	/	/	/	6	7	/	/	/	/	/	/
Sonia M. D. Brucki et al. [135]	RS	Brazil	63	50.8	60	1	/	/	/	5	25	/	/	/	/	/	17	/	1
Denise Battaglini et al. [136]	RS	Italy	94	78.7	61.6	/	/	/	/	2	3	/	/	/	/	/	2	/	/
Sofía Portela- Sánchez et al. [137]	PS	Spain	71	70.4	69	/	/	/	3	6	16	/	/	/	/	/	15	/	/
Mehran Ghaffari et al. [138]	RS	Iran	361	59.3	61.9	109	34	41	4	10	8	174	69	69	/	/	11	/	1
David T. Chuang et al. [139]	RS	USA	56	43	69.2	7	/	/	2	6	18	/	/	/	3	25	/	/	/
Man Amanat et al. [140]	PS *	Iran	873	63.7	60.71	110	104	/	/	/	/	217	/	/	/	/	/	/	/
Siyuan Fan et al. [141]	RS	China	86	62.8	66.6	8	6	/	/	/	6	15	/	/	/	/	/	/	/
Giovanna Travi et al. [142]	RS	Italy	901	51.7	64	39	/	/	/	19	53	/	/	/	/	/	/	5	/
Hatice Yuksel et al. [143]	RS	Turkey	204	58.5	67.22	28	32	139	/	27	22	/	/	/	/	132	/	/	/
Verena Rass et al. [144]	PS,cohort study *	Austria	135	61	56	/	/	/	/	1	1	/	1	/	/	/	2	/	1
Marco H. Carcamo Garcia et al. [145]	PS, CSS	Peru	199	43	43	143	68	/	/	1	/	92	80	81	17	7	/	/	/
Juan Carlos García-Moncó et al. [146]	PS, CSS	Spain	35	71	66	3	/	/	/	2	3	/	/	/	/	/	/	/	/
David García-Azorín et al. [147]	RS *	Spain	233	54.9	61.1	30	/	55	/	27	63	/	41	/	/	/	/	/	/
David García- Azorí et al. [148]	RS,cohort study	Spain	576	56.6	67.19	137	11	98	/	3	/	139	146	/	/	/	/	/	/
Ummehan Ermis et al. [149]	PS, CSS	Germany	53	60	63	11	6	/	/	/	/	/	14	8	/	/	2	/	/
Andrea Giorgianni et al. [150]	RS	Italy	26	46.1	70.6	1	3	/	/	/	/	/	/	/	/	/	/	1	/
A. Patel et al. [151]	RS	UK	141	58.9	45.6	/	/	/	/	/	/	93	80	89	/	/	/	/	/
Fatemeh Sadat Mirfazeli et al. [152]	RS	Iran	201	100	51.84	80	/	/	/	4	2	/	68	66	/	/	/	/	/
Fazilet Altin et al. [153]	PS *	Turkey	81	50.6	54.16	/	/	/	/	/	/	/	50	22	/	/	/	/	/
Kuganathan Ramasamy et al. [154]	RS	Malaysia	145	62.1	43	8	1	/	/	/	/	/	31	34	/	/	/	/	/
Alberto Paderno et al. [155]	PS,cohort study	Italy	151	37	45	/	/	/	/	/	/	/	126	135	/	/	/	/	/
Marlene M. Speth et al. [156]	PS, CSS	USA	103	48.5	46.8	/	/	/	/	/	/	/	63	67	/	/	/	/	/
Kunal Thakur et al. [157]	PS	India	250	57.6	/	124	/	/	/	/	/	98	179	79	/	/	/	/	/
Müge Özçelik Korkmaz et al. [158]	PS,cohort study	Turkey	116	50	57.24	43	37	/	/	/	/	/	44	48	/	/	/	/	/
Elif Elibol et al. [159]	RS	Turkey	155	42.2	36.3	/	/	/	/	/	/	/	55	25	/	/	/	/	/
Jerome R. Lechien et al. [160]	RS	France	86	34.9	41.7	42	/	/	/	/	/	30	53	/	/	/	/	/	/
G. O’Sullivan et al. [161]	RS, case–control study	Ireland	102	23	44.1	36	/	/	/	/	/	30	4	4	/	/	/	/	/
Edith L. Graham et al. [162]	PS	USA	50	34	43.7	32	20	/	/	/	/	30	37	32	9	/	/	/	/
Kate Gregorevic et al. [163]	RS	Australia	106	40.6	84.3	7	7	/	/	/	/	/	/	/	/	/	/	/	/
Hao Lv et al. [164]	RS *	China	196	44.9	50.6	11	/	/	/	/	/	26	16	9	/	/	/	/	/
Agathe Nouchi et al. [165]	CSS	France	390	51.8	50.5	148	/	/	/	/	/	/	129	130	/	/	/	/	/
Shima Shahjouei et al. [166]	PS *	USA	432	57.6	65.7	/	/	/	91	/	323	/	/	/	/	/	/	/	/
Patricia Bustos et al. [167]	RS	Chile	458	47.4	46.35	216	/	/	/	/	/	281	/	/	/	/	/	/	/
Adnan A. Mubaraki et al. [168]	RS	Kingdom of Saudi Arabia	1022	60.9	/	520	/	/	/	/	/	605	542	525	/	/	/	/	/
Egehan Salepci et al. [169]	CSS	Turkey	223	50.7	51	59	5	/	/	/	/	113	71	77	/	/	/	/	/
J. Zhang et al. [170]	RS,cohort study	China	663	48.4	55.6	20	23	/	/	/	/	63	/	/	/	/	/	/	/
Alberto Paderno et al. [171]	CSS	Italy	508	56	55	198	/	/	/	/	/	/	283	321	/	/	/	/	/
Izquierdo-Domínguez A et al. [172]	PS, CSS *	Spain	846	52.7	56.8	/	/	/	/	/	/	/	454	442	/	/	/	/	/
Alma Tostmann et al. [173]	RS	The Netherlands	90	21.1	/	64	/	/	/	/	/	57	37	/	/	/	/	/	/
Maria Rosaria Barillari et al. [174]	RS *	Italy	179	50.3	41	73	/	/	/	/	/	84	/	/	/	/	/	/	/
Elisabeth Ninchritz-Becerra et al. [175]	PS	Spain	1043	36.4	39	782	/	/	/	/	/	719	826	718	/	/	/	/	/
Brian E. Dixon et al. [176]	RS	USA	368	44.4	/	/	/	/	/	/	/	117	94	105	/	/	/	/	/
Eman M. Khedr et al. [177]	RS	Egypt	55	50.9	51.5	21	9	/	/	/	/	/	/	/	/	/	2	2	2
Pınar Sayın et al. [178]	RS	Turkey	52	69.2	61.32	/	/	/	/	/	/	/	21	19	/	/	/	/	/
Eric J. Chow et al. [179]	RS	USA	48	22.9	43	20	/	/	/	/	/	29	/	/	/	/	/	/	/
Altunisik E et al. [180]	RS	Turkey	51	56.9	52.78	9	7	/	/	/	/	/	3	3	/	4	/	/	/

*RS* Retrospective study, *CS* case series, *PS* prospective study, *CSS* cross-sectional study

*Multicenter

Myalgia(33%; 95%CI 0.30–0.37; I^2^ = 99.17%) (86 studies), smell impairment(33%; 95%CI 0.28–0.38; I^2^ = 99.40%) (106 studies), taste dysfunction(33%; 95%CI 0.27 -0.39; I^2^ = 99.09%) (80 studies) and altered mental status(32%; 95%CI 0.22–0.43; I^2^ = 99.06%) (18 studies) were the most common neurological manifestations of COVID-19. Headache(29%; 95%CI 0.25–0.33; I^2^ = 99.42%) (123 studies), encephalopathy(26%; 95%CI 0.16–0.38; I^2^ = 99.31%) (23 studies), alteration of consciousness(13%; 95%CI 0.08–0.19; I^2^ = 98.10%) (19 studies), stroke(12%; 95%CI 0.08–0.16; I^2^ = 98.95%) (47 studies), dizziness(10%; 95%CI 0.08–0.13; I^2^ = 96.45%) (50 studies), vision impairment(6%; 95%CI 0.03–0.09; I^2^ = 86.82%) (14 studies), intracerebral haemorrhage(5%; 95%CI 0.03–0.09; I^2^ = 95.60%) (21 studies) and seizure(4%; 95%CI 0.02–0.05; I^2^ = 98.15%) (47 studies) were the next most common. The less common neurological manifestations were encephalitis(2%; 95%CI 0.01–0.03; I^2^ = 90.36%) (14 studies) and Guillan-Barré Syndrome (GBS) (1%; 95%CI 0.00–0.03; I^2^ = 89.48%) (12 studies).

In all the included literature, 16 studies grouped and analyzed the neurological characteristics according to the severity of COVID-19. Through meta-analysis, we concluded that there was no significant difference in the incidence of headache(16%;95%CI 0.11–0.22; I^2^ = 94.05% VS 16%;95%CI 0.10–0.23; I^2^ = 97.77%) between the severe group and the non-severe group, and the incidence of dizziness(12%;95%CI 0.07–0.18; I^2^ = 88.28% VS 9%;95%CI 0.04 − 0.16; I^2^ = 96.05%) and seizure(3%;95%CI 0.01–0.06; I^2^ = 83.80% VS 1%;95%CI 0.00–0.03; I^2^ = 86.05%)was higher, while myalgia(21%;95%CI 0.13–0.29; I^2^ = 97.82% VS 24%;95%CI 0.16–0.32; I^2^ = 98.39%), smell impairment(8%;95%CI 0.05–0.13; I^2^ = 87.77% VS 13%;95%CI 0.08–0.18; I^2^ = 92.34%) and taste dysfunction(9%;95%CI 0.05–0.14; I^2^ = 90.24% VS 14%;95%CI 0.10–0.20; I^2^ = 93.21%) were less. Table 2 shows the details and characteristics of the 16 included studies divided into severe and non-severe groups.

**Table 2:** The details and characteristics of the 16 included studies divided into severe and non-severe groups

Study	Severe							Non-severe						
	Case	Headache	Dizziness	Seizure	Myalgia	Smell impairment	Taste dysfunction	Case	Headache	Dizziness	Seizure	Myalgia	Smell impairment	Taste dysfunction
Ling Mao et al. [8]	88	15	17	1	/	3	3	126	13	19	/	/	8	9
Antoine Guilmot et al. [42]	9	/	/	1	/	/	/	6	/	/	1	/	2	/
Nitesh Gupta et al. [68]	32	13	/	/	/	/	/	168	9	/	/	/	/	/
François Lersy et al. [84]	47	1	/	3	/	/	/	11	2	/	3	/	/	/
Rajesh Benny et al. [85]	47	8	/	9	/	7	2	53	13	/	9	/	3	6
Daniel Schonfeld et al. [94]	5652	1168	/	60	1002	380	320	41,703	12,517	/	223	9572	5893	4508
Caizheng Yu et al. [110]	864	/	/	/	31	/	/	799	/	/	/	26	/	/
Shenae Samuels et al. [111]	147	21	/	/	30	/	/	346	26	/	3	86	/	/
Eric M. Liotta et al. [113]	134	43	40	/	56	11	17	375	149	111	4	172	47	64
Carlos Manuel Romero-Sánchez et al. [129]	329	38	17	4	44	9	13	512	81	34	2	101	32	39
Xudong He et al. [132]	23	3	4	/	8	/	/	54	1	5	/	10	/	/
Mehran Ghaffari et al. [138]	233	60	22	7	101	42	41	128	49	12	3	73	27	28
Man Amanat et al. [140]	299	24	29	/	75	/	/	574	86	75	/	142	/	/
Egehan Salepci et al. [169]	45	11	4	/	/	6	12	178	48	1	/	/	65	65
J. Zhang et al. [170]	409	13	21	/	30	/	/	254	7	2	/	33	/	/
Altunisik E et al.(180)	11	4	2	/	/	/	/	40	5	5	/	/	3	3

Of all the included literatures, 16 studies grouped neurologic features based on COVID-19 severity. We focused on five neurologic manifestations of headache,dizziness,seizure,myalgia, smell impairment and taste dysfunction

**Fig. 2:**
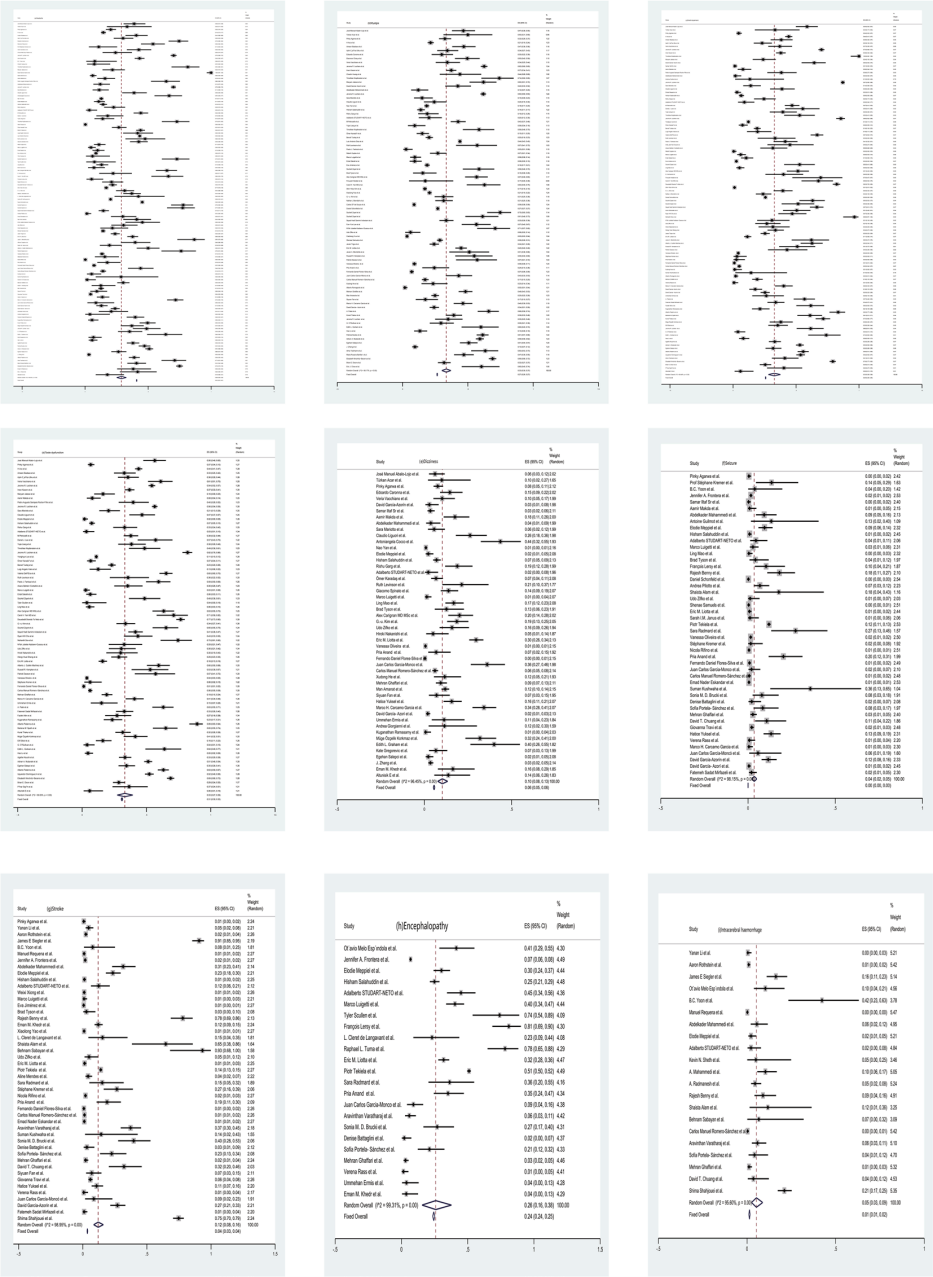

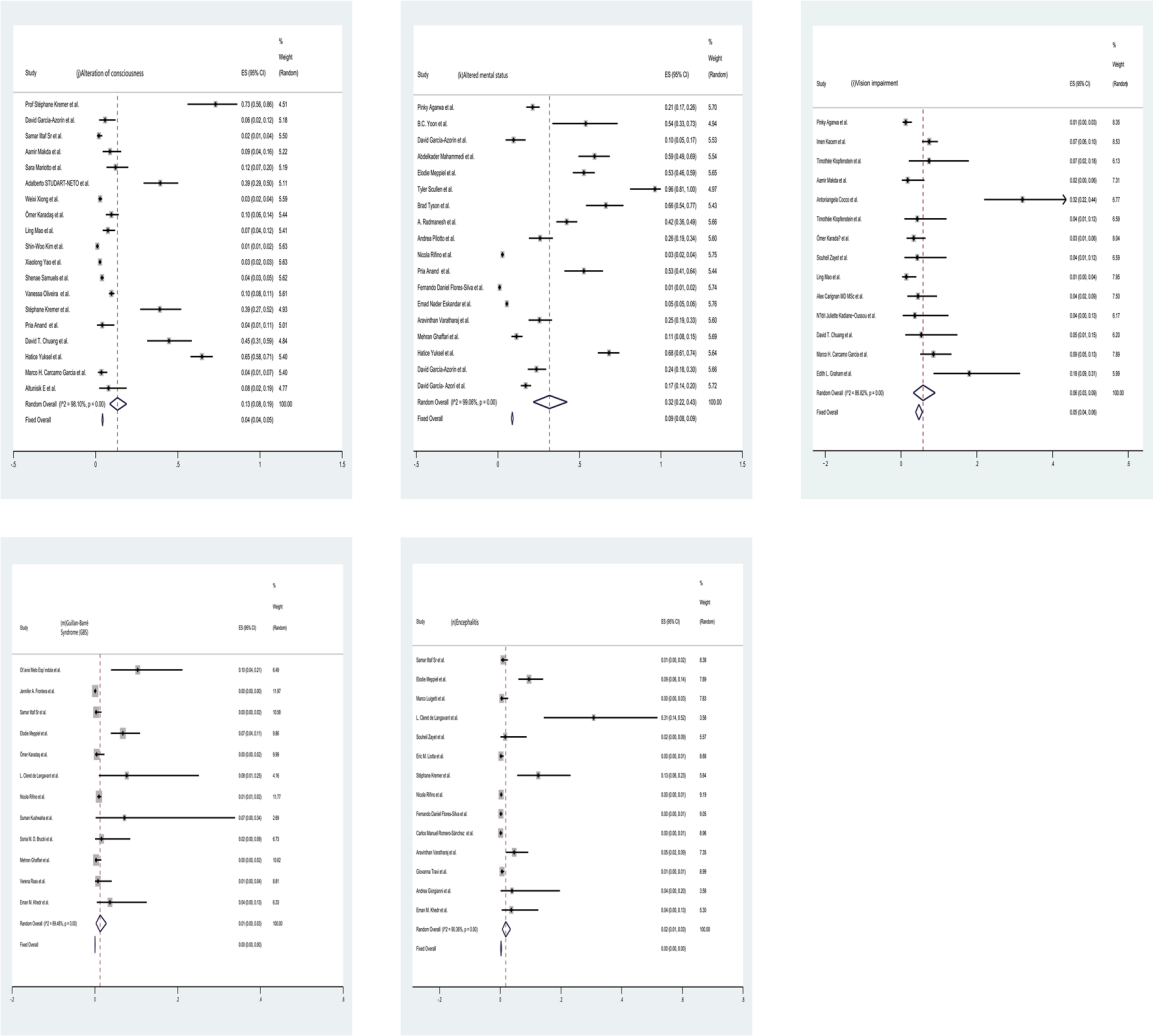
Forest maps of neurological manifestations. **a** Headache, **b** myalgia, **c** smell impairment, **d** taste dysfunction, **e **dizziness, **f** seizure, **g** stroke, **h** encephalopathy, **i** intracerebral haemorrhage, **j** alteration of consciousness, **k** altered mental status, **l** vision impairment, **m** Guillan-Barré Syndrome (GBS) and **n** encephalitis. After the heterogeneity test, the results in figures **a**–**n** all suggest that there was significant heterogeneity among the selected literature in this study

**Fig. 3:**
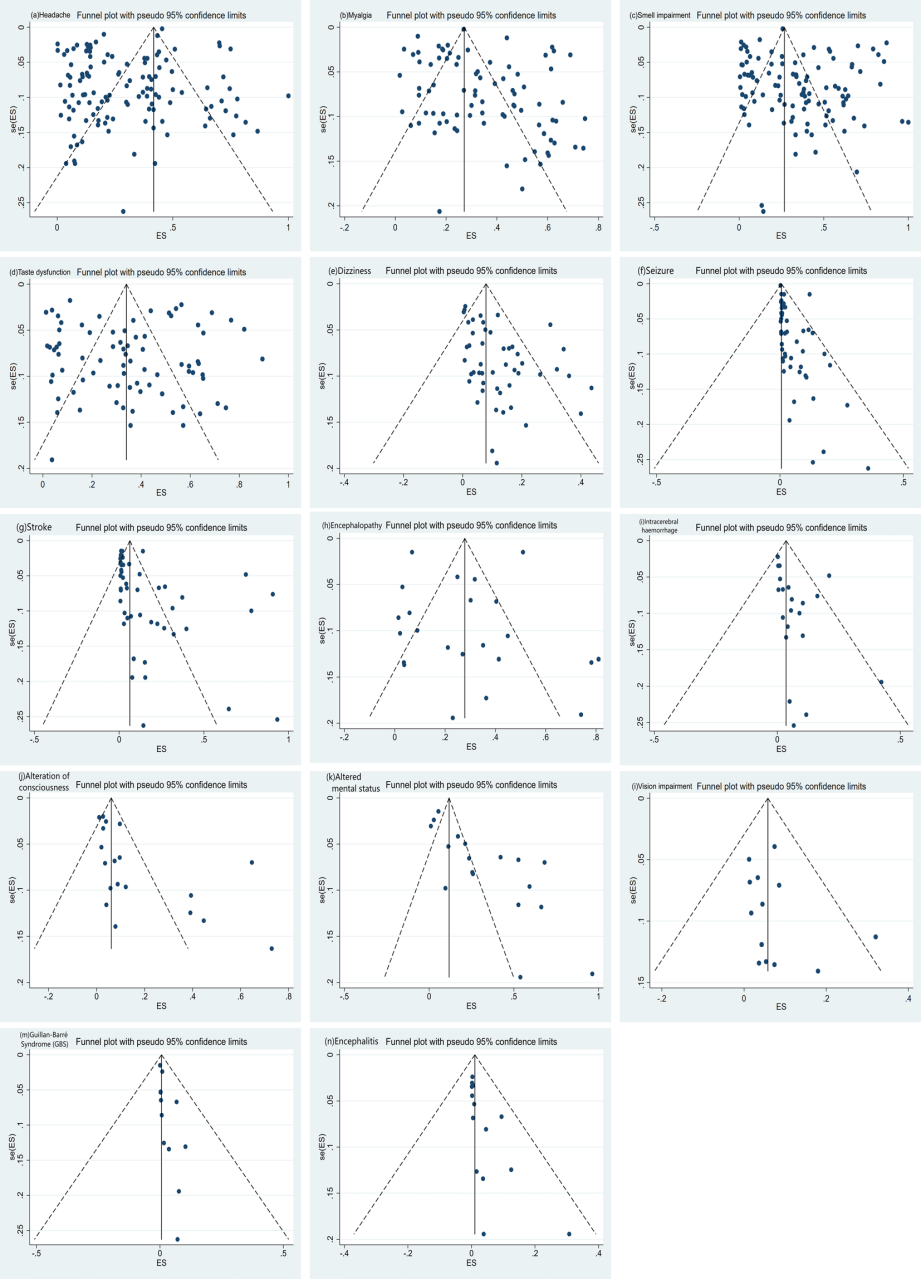
Funnel plots. **a** Headache, **b** myalgia, **c** smell impairment, **d** taste dysfunction, **e** dizziness, **f** seizure, **g** stroke, **h** encephalopathy, **i** intracerebral haemorrhage, **j** alteration of consciousness, **k** altered mental status, **l** vision impairment, **m** Guillan-Barré Syndrome (GBS) and **n** encephalitis. After the bias test, the visual symmetry of the funnel plot and Egger's test showed that the publication of myalgia, taste dysfunction, encephalopathy and vision impairment was unbiased

**Fig. 4:**
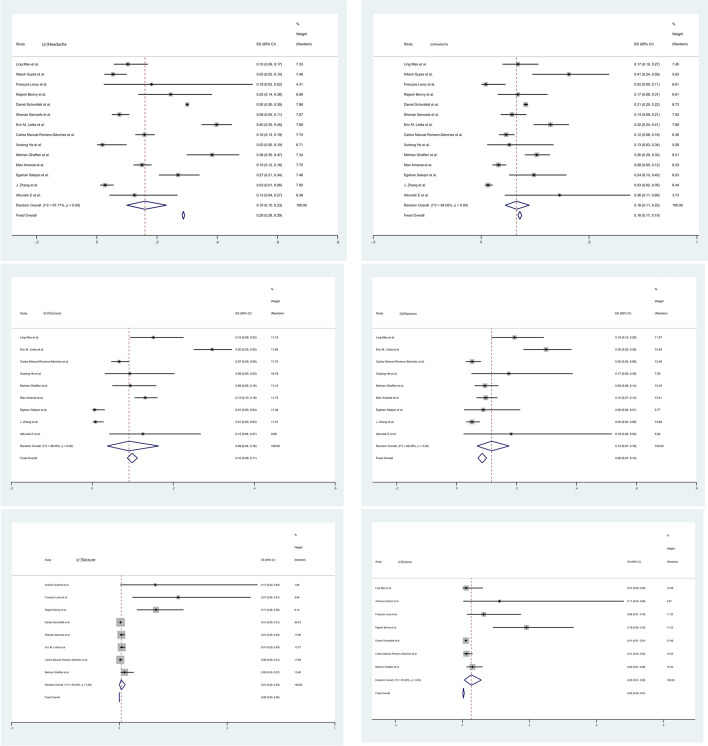

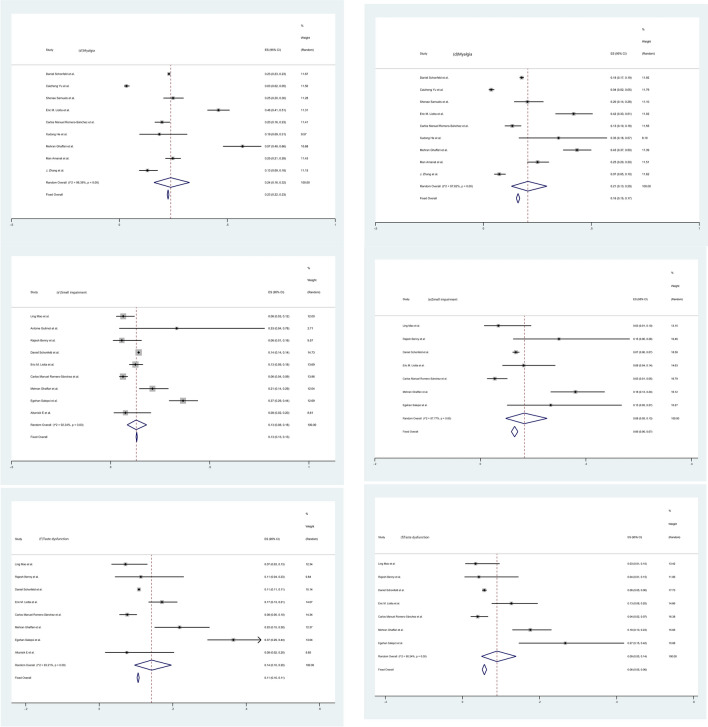
Forest plots of the severe and non-severe groups. **a** Headache, **b** dizziness, **c** seizure, **d** myalgia, **e** smell impairment, **f** taste dysfunction in severe group and **a’** headache, **b’** dizziness, **c’** seizure, **d’** myalgia, **e’** smell impairment, **f’** taste dysfunction in the non-severe group. After the heterogeneity test, the results in figures **a**/**a’** to **f**/**f’** all suggest that there was significant heterogeneity among the selected literature in this study.)

**Fig. 5:**
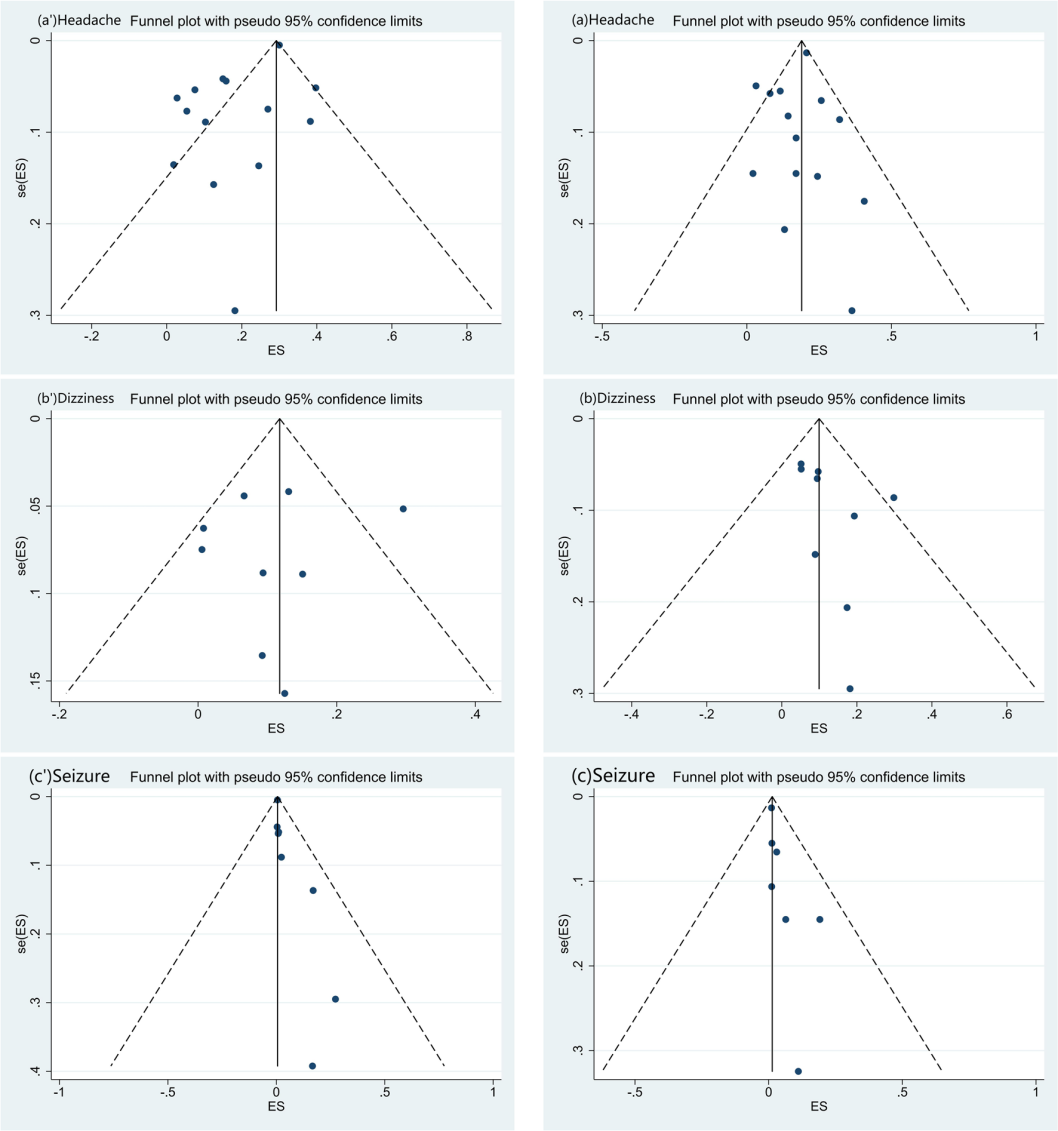

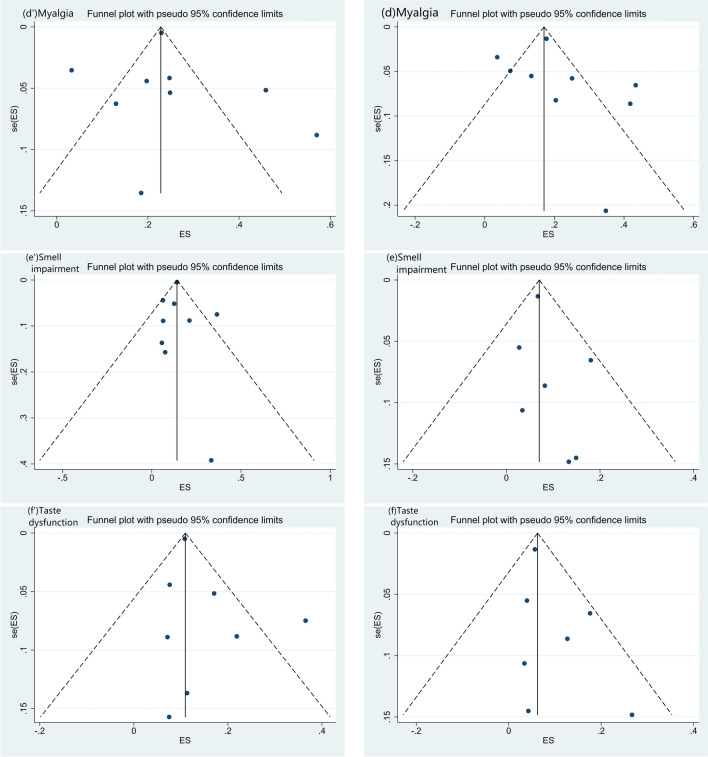
Funnel plots for the severe and non-severe groups. **a** Headache, **b** dizziness, **c **seizure, **d** myalgia, **e** smell impairment, **f** taste dysfunction in severe group and **a’** headache, **b’** dizziness, **c’** seizure, **d’** myalgia, **e’** smell impairment, **f’** taste dysfunction in non-severe group. Funnel plot results and Egger's test showed that there was no publication bias in dizziness, seizure, myalgia, smell impairment, and taste dysfunction in both groups

Included studies had good methodological quality (Figs. 2, 3, 4, 5).

## Discussion

According to our meta-analysis, the most common neurological manifestations of COVID-19 patients were myalgia (33%), smell impairment (33%), taste dysfunction (33%) and altered mental status (32%). It can be seen that both the central nervous system and peripheral nervous system will be involved after patients are infected with SARS-CoV-2. At the same time, considering the difference between symptoms and diseases, our study can conclude that the most common central nervous system symptoms are altered mental status (32%), headache (29%), alteration of consciousness (13%), and dizziness (10%). The most common central nervous system (CNS) disorders were encephalopathy (26%), stroke (12%), intracerebral haemorrhage (5%), seizure (4%), and encephalitis (2%). The most common symptoms of peripheral nervous system (PNS) impairment were myalgia (33%), smell impairment (33%), taste dysfunction (33%) and vision impairment (6%). Guillan-Barre Syndrome (GBS) occurs in approximately 1% of peripheral nervous system disorder.

In addition, COVID-19 involvement in the peripheral nervous system includes other manifestations, such as cranial neuropathy and neuromuscular joint disease, etc. However, since only a few studies have reported such cases, and most of these studies were case reports, these less frequent COVID-19 peripheral nervous system manifestations were not included in this study. In the future, as more and more of these studies are reported, we can conduct further research.

Among 168 studies that were finally included in the meta-analysis, 123 studies discussed the incidence of headache, 106 studies discussed smell impairment, 86 studies discussed myalgia, and 80 studies discussed taste dysfunction. In our opinion, this also indirectly indicates that COVID-19 patients have symptoms of headache, smell impairment, myalgia and smell impairment earlier and have more cases, which is worthy of further study and discussion, to provide a diagnosis and treatment direction for early intervention in the future.

In 16 of the included studies, patients were divided into severe and non-severe groups for detailed analysis according to the severity of COVID-19 infection in patients. We found that the most studied neurological manifestations in this literature were headache, dizziness, seizure, myalgia, smell impairment and taste dysfunction. Our results showed that there was no significant difference in the incidence of headache between the two groups, both at 16%; the incidence of dizziness and seizure in the severe COVID-19 group was higher than that in the non-severe group (12% VS 9%, 3% VS 1%, respectively). The incidence of myalgia, smell impairment and taste dysfunction in the severe COVID-19 group was lower than that in the non-severe group, which was 21% VS 24%, 8% VS 13%, 9% VS 14%, respectively. However, considering that the severity of COVID-19 disease is not classified the same according to different prevalence periods of COVID-19 and its prevalence in different countries, our statistical data may be biased to a certain extent, and the statistical results may lack a certain scientific nature. At the same time, there are still limited studies on various neurologic characteristics of COVID-19 under different severity, which deserves more research and exploration.

We also found that altered mental status, encephalopathy, and alteration of consciousness were reported separately, but they may be related to each other. Through reading different literature, we found that different researchers have different definitions of the above three aspects. Josef Anrather et al. [181] argue that altered mental status (e.g., confusion, disorientation, emotional restlessness and lethargy) can be collectively referred to as encephalopathy. Emad Nader Eskandar et al. [130] suggested that if there was evidence of cognitive impairment (e.g., confusion, disorientation, agitation or delirium) or impaired arousal (e.g., lethargy or dullness), the patient can be included in the altered mental status cohort. In another study, altered mental status was defined as including personality, behavior, cognition, and consciousness changes; encephalopathy, encephalitis; catatonia, mania, anxiety or depression, etc. [131]. We recognize that there may be overlapped parts among altered mental status, encephalopathy and alteration of consciousness, but they can all indicate that the central nervous system of patients is damaged, so their clinical reference value is not affected.

## Associations between neurologic manifestations and mechanisms of nervous system injury in patients with COVID-19

ACE2 has been proved to be a functional receptor of SARS-CoV-2, which binds to the ACE2 receptor through its Spike (S) protein C-terminal domain (CTD) [182]. The expression profile of ACE2 is very extensive, and it is expressed in various regions of the human brain, such as the ventricle, motor cortex and posterior cingulum gyrus, middle temporal gyrus, substantial nigra, olfactory bulb, contralateral medulla oblongata, nucleus solitaries, vagus nerve, neurons, astrocytes, microglia and oligodendroglia, etc. [183, 184]. Therefore, the nervous system is at risk of SARS-COV-2 infection. The mechanism of nervous system injury in COVID-19 patients includes cross-neuronal hypothesis, homogenous transmission and BBB transmission, hypoxia, inflammatory response and hypercoagulability and immune mechanism as well. In the following sections, we will analyze and discuss the relationship between the neurological manifestations we have studied and the corresponding mechanisms of neurological damage.

### Altered mental status and alteration of consciousness

The altered mental status and alteration of consciousness of COVID-19 patients may be a systemic consequence of the over-activation of the body's immune response or may be caused by the direct invasion of the nerves by SARS-COV2 [185, 186]. Hypoxia, organ dysfunction, the need for large doses of sedatives, and prolonged isolation may also contribute to consciousness changes [181].

### Headache and dizziness

Headaches and dizziness can be caused by a variety of causes. One reason may be the large and rapid increase of inflammatory cytokines, including IL-1, IL-6, and TNFα, after infection. It can also cause pain if the virus invades the nervous system directly and damages the nerve. Hypoxia leads to the accumulation of acid in brain cells, swelling, and interstitial edema, resulting in cerebral vasodilation or cerebral blood flow obstruction, etc., which is also considered as one of the potential mechanisms of causing headache [185].

### Myalgia

Because there are ACE2 receptors in skeletal muscle [187], direct cytotoxicity caused by the interaction between SARS-CoV-2 and ACE2 in skeletal muscle should be considered. Secondly, muscle injury may be related to the harmful immune response mediated by infection and the increase of pro-inflammatory cytokines [8]. Elevated concentrations of C-reactive protein and D-dimer in the blood may induce the expression of a strong immune response, leading to direct immune-mediated nerve and muscle injury [188]. At the same time, prolonged mechanical ventilation or the use of neuromuscular blockers in patients with severe COVID-19 infection is also closely related to the progression of critical myopathy [189].

### Smell impairment and taste dysfunction

Studies have shown that the anosmia of most COVID-19 patients has nothing to do with rhinorrhea or nasal obstruction, and may be related to the transmission of the virus through olfactory nerve epithelial cells and further invasion of the olfactory bulb and central nervous system [43, 190]. It has been reported that higher levels of angiotensin can induce the apoptosis effect of neural stem cells [191], reducing the number of nerve cells migrating to the olfactory bulb, resulting in reduced replacement of new neurons in the olfactory bulb, and interference with the smell. Animal experiments have shown that SARS-CoV-2 may invade the brain retrograde along with taste and trigeminal nerve pathways in the early stage of infection, causing lesions such as taste disturbance [192].

### Encephalopathy and encephalitis

Patients with COVID-19 may develop many types of encephalopathy. The combination of SARS-CoV-2 virus and ACE2 receptor increases peripheral vascular resistance, leading to a significant and rapid increase in blood pressure, triggering a cascade reaction that leads to the destruction of blood–brain barrier integrity, cerebral hyperperfusion, and cerebral edema, leading to the development of hypertensive encephalopathy [193, 194]. Hypoxia, insufficient energy supply, or overall insufficiency after severe brain injury can progress to hypoxic-ischemic encephalopathy. Steatosis, liver function damage, or cardiovascular dysfunction after COVID-19 infection may induce the occurrence of hepatic encephalopathy, which may be related to direct liver damage of SARS-CoV-2 or the side effects of some drugs [195, 196]. In addition, recent studies have found an increased incidence of acute renal damage after SARS-CoV-2, and the pathophysiological mechanism remains unclear, which may also be caused by an inflammatory response, ACE/ACE2 imbalance, or dysfunction of other organs (such as the heart). When patients have renal insufficiency, the body cannot completely discharge toxins and regulate the concentration of cytokines normally, and the brain homeostasis will be destroyed, leading to uremic encephalopathy [197]. When SARS-CoV-2 enters the human body through ACE2, the human body immediately generates an immune response. When the virus invades the brain, it will cause immune damage and lead to the attack of encephalitis [198]. Encephalitis may also be associated with blood–brain barrier disruption and cytokine surges [199].

### Stroke and intracerebral hemorrhage

SARS-CoV-2 directly invades the nerve through ACE2 receptors and deregulation of blood pressure is the potential mechanism of COVID-19 stroke [200, 201]. Continuous hypoxia in COVID-19 patients will eventually lead to disorders in neurotransmitter metabolism and mitochondrial failure, causing irreversible nerve damage and increasing the risk of stroke [202]. Studies have shown that increased inflammatory markers such as IL-2, IL-6, macrophage inflammatory protein 1-α or coagulation dysfunction in patients with severe COVID-19 may increase the likelihood of stroke compared with moderate patients [200]. After the SARS-CoV-2 virus binds to the ACE2 receptor, it can lead to increased blood pressure, which may lead to hypertensive crisis in severe cases and increase the risk of intracranial hemorrhage [188].

### Seizure

The process of SARS-CoV-2 virus replication in host cells disrupts neuronal function and manifests as seizures, convulsions, and loss of consciousness [203]. The increase of pro-inflammatory mediators may contribute to epileptic seizures, which in turn enhance the production of cytokines such as IL-1B and TNFα [204]. After the destruction of the blood–brain barrier, inflammatory factors and viral particles flood into the central nervous system, leading to brain damage that can lead to epileptic seizures [188].

### Guillan-Barré Syndrome (GBS)

Guillain–Barre syndrome (GBS) is an increasing occurrence in patients with COVID-19, a typical viral autoimmune nervous system disease caused by a strong immune response, which includes a range of polyneuropathy characterized by acute motor weakness, mild to moderate paresthesia, cranial nerve involvement, and muscle or nerve root pain [205]. At present, large sample studies on GBS are still lacking, and most researchers express their views in the form of case reports.

## Biomarkers reflecting nervous system damage in COVID-19 patients

A prospective study showed that total Tau, GFAP, and NFL protein levels in cerebrospinal fluid were elevated in 63, 37, and 16% of patients, respectively, and NFL protein was associated with disease severity, duration of intensive care, and level of consciousness [206]. Multiple studies on COVID-19 patients with ischemic stroke have shown that the neutrophil–lymphocyte ratio (NLR) is increased in 90% of the patients [207], CRP is increased in over 90% of the patients [208], and serum ferritin is also increased [209], in which serum ferritin can also predict the degree of nerve injury in patients with acute ischemic stroke [210]. There was a significant correlation between the decrease of interleukin-6 level and the improvement of olfaction and taste function in COVID-19 patients [211]. Elevated serum nerve filament light chain (SNFL) levels in critically ill patients with COVID-19 are closely associated with poor prognosis [212].

## Examination methods reflecting nervous system damage in COVID-19 patients

In our study, stroke incidence was 12%, intracerebral hemorrhage 5%, encephalopathy 26% and encephalitis 2%. Studies have shown that patients with COVID-19 with acute neuroimaging abnormalities are more likely to have an ischemic stroke. Neuroimaging features of these patients are not invariable but are predominated by acute ischemic infarction, intracranial hemorrhage, and leukoencephalopathy [81]. COVID-19 is an independent risk factor for acute ischemic stroke and a valid indicator of poor prognosis. Meningitis and encephalitis are not very common [213, 214]. In one study, continuous EEG monitoring was performed in 11 of 16 patients with COVID-19, most of whom presented with nonspecific encephalopathy [215]. At the same time, some studies also found significant structural changes in the olfactory nerve, olfactory bulb, olfactory cortex and other olfactory pathways in MRI examination of COVID-19 patients, suggesting that SARS-CoV-2 may enter the central nervous system through the olfactory bulb mediated cross-neuronal pathway [94, 95]. The relationship between COVID-19 imaging changes and neurological symptoms requires more research.

## Prognosis of nervous system injury in COVID-19 patients

Nervous system damage is closely associated with the mortality of SARS-COV-2 infection, and whether the neurological symptoms are reversible is not yet clear. Patients with COVID-19 who require ICU admission for neurological problems or develop neurological dysfunction in the ICU have significantly increased mortality [216]. In one animal study, 4 out of 14 infected mice developed significant respiratory distress and neurological symptoms 2 days after infection, and only the mice showing neurological symptoms died, suggesting that neurological involvement may be a cause of death [217]. Some studies have confirmed that recovery from acute SARS-CoV-2 infection does not completely clear the virus, and has been found to have a higher potential risk for long-term residual neuropsychiatric and neuroscientist impairments, including depression, obsessive–compulsive disorder, psychosis, Parkinson's disease and Alzheimer's disease [218]. An experimental animal study has also shown that coronavirus can persist in the central nervous system of its host [219]. The study results of Helms et al. showed that 36% of severe COVID-19 survivors developed Dysfunction Syndrome [220], and some studies also reported that patients with severe dysfunction after the acute phase had significant recovery after active neurological rehabilitation [221]. In a prospective study, 68.33% of patients developed neurological symptoms during infection with SARS-CoV-2, and 50% recovered 3 months after infection but still had neurological symptoms [213]. Imaging results of 60 patients after recovery of COVID-19 neurological symptoms suggest that the microstructure and functional brain integrity of the brain may be damaged during the rehabilitation phase, which may require long-term neurological and neuroimaging follow-up [214].

## Advantages and disadvantages of this study

Our meta-analysis included 168 articles (n = 292,693) detailing the various neurologic symptoms common in COVID-19 patients and providing a comprehensive view of the neurologic symptoms of COVID-19. The results were comprehensive. We started the discussion of the clinical manifestations and analyzed the evidence, possible mechanism and prognosis of the nervous system injury caused by SARS-CoV-2. We also discussed the biomarkers and examination methods of nervous system injury caused by SARS-COV-2, providing some valuable suggestions for early identification, monitoring, screening, diagnosis and follow-up of nervous system injury and poor prognosis in patients with COVID-19 and potential targets for future clinical intervention strategies.

This study also has some limitations. Firstly, most of the included literature was retrospective studies, which may cause some potential bias. Secondly, this study failed to provide an analysis of the correlation between various neurological manifestations and disease severity and mortality. The question of which neurological manifestations are the most insidious and which are the most difficult to recover from is currently unanswered, and more findings will be needed in the future. Finally, the high degree of heterogeneity in our study may be due to differences in patient race selection, disease severity, comorbidities, only a few studies specifically assessed neurological symptoms, differences in the number of patients in different studies, or differences in publication bias and study methodology.

## Conclusion

In conclusion, our study suggests that nervous system expression in COVID-19 is diverse and pervasive but easily underestimated. Therefore, the long-term pathophysiological results of SARS-COV-2 neuropathy should be of serious concern to us. At the same time, summarizing the entire symptom spectrum, biomarkers and examination methods of the disease is conducive to predicting the severity of neurological impairment and providing better suggestions for preventing misdiagnosis, early diagnosis, preventing disease transmission, early intervention treatment, evaluation of therapeutic effect and follow-up. In the future, more clinical and experimental studies should be carried out to provide strong evidence to support clinical practice, and further, explore the role of nervous system symptoms in the incidence of COVID-19 and its possible mechanism, to reduce mortality, reduce disease severity, control disease progression and prevent possible long-term nervous system complications.

## Data Availability

Not applicable.
